# Challenges and Successful Pedagogical Strategies: Experiences from Six Swedish Students with Blindness and Autism in Different School Settings

**DOI:** 10.1007/s10803-017-3360-5

**Published:** 2017-10-27

**Authors:** Kim de Verdier, Elisabeth Fernell, Ulla Ek

**Affiliations:** 1 Department of Special Education, Stockholm University, 106 91 Stockholm, Sweden; 2 National Agency for Special Needs Education and Schools, P. O. Box 121 61, 102 26 Stockholm, Sweden; 3 Gillberg Neuropsychiatry Centre, Institute of Neuroscience and Physiology, University of Gothenburg, Kungsgatan 12, 411 19 Gothenburg, Sweden; 4 Department of Special Education, Stockholm University, 106 91 Stockholm, Sweden

**Keywords:** Autism, Blindness, Children, School, Executive functions, Support, Education

## Abstract

The prevalence of autism in children with blindness is much higher than in the general population. There are many challenges regarding the school situation for children with this complex dual disability. This study explored challenges and successful strategies in school for a sample of six Swedish children with blindness and autism, with and without intellectual disability, through qualitative interviews with students, teachers and parents. All students displayed executive functioning deficits, and the teaching situation entailed several challenges. Our research points to the importance of adopting evidence-based practices for ASD, but adapted according to the students lack of vision. For this to be possible, close collaboration between teachers, parents and specialists in the field of visual impairment and autism is necessary.

## Introduction

Blindness in combination with autism spectrum disorder (ASD) is a rare dual disability that entails many challenges for a child’s development. The prevalence of blindness, in different parts of the world, varies between 0.3–0.4/1000 and 1.0–1.5/1000 children (Gilbert 2007). There is an increasing awareness that a large proportion of the population of blind children also has ASD. While the prevalence of ASD in the general child population is around 1% (Lazoff et al. 2010), research has shown that among children with blindness, regardless of etiology, at least 30% meet the criteria for ASD (Cass 1998; Hobson et al. 1999; de Verdier et al. 2017). Jure et al. (2016) reported even higher numbers; 50% overall, and in children with retinopathy of prematurity (ROP) and congenital blindness, 65 and 72%, respectively. The co-occurrence of blindness and ASD in specific etiological subgroups has been described in several studies. For example, in a Swedish population-based study of extremely preterm-born children with ROP, nearly two-thirds of the children with total blindness had ASD, and all of them also had an intellectual disability (ID) (Ek et al. 1998; Jacobson et al. 1998). Optic Nerve Hypoplasia (ONH) has also been found to be strongly associated with ASD (Ek et al. 2005; Garcia-Filion et al. 2008; Parr et al. 2010), as well as anophthalmia/microophthalmia, which has been reported to co-exist with ASD in genetic cranio-facial malformation syndromes, including CHARGE (Blyth and Baralle 2011; Pushker et al. 2013). Consequently, autism is currently one of the most commonly reported coexisting developmental disorders in children with blindness (Ek 2010).

Blindness in itself has a significant impact on a child’s development, and if the child has additional disabilities the situation is even more challenging. Children with blindness and ASD, with and without ID, have complex needs, and the environment has a great responsibility to interpret and meet these needs to promote development and learning. For many of these children, the surrounding world can be very confusing. A constant mix of sensory stimuli can be overwhelming and even impede learning (Gense and Gense 2005). The child’s multiple processing problems can also create conflicts regarding the choice of pedagogical approach in the teaching situation (Jordan 2005). It is important to understand and consider the child’s needs related to both the visual impairment (VI) and the ASD, and how the combination influences the child’s learning, in shaping the pedagogical methods (Gense and Gense 2005). If either one of the disabilities is overlooked, the method is likely to fail (Gibbons 2005). However, while there is a vast body of research on evidence-based practices for sighted children with ASD (Odom, 2011), very little is reported in the literature about evidence-based practices for children with ASD and blindness (Gense and Gense 2011). Many of the methods applied for children with ASD, such as Treatment and Education of Autistic and related Communication handicapped Children (TEACCH), Applied Behavior Analysis (ABA), the Picture Exchange Communication System (PECS), visual modelling and Comic strip conversation, build strongly on images or other visual input. The general principle of the method can often be applied, but the realization and adaptation of this principle into a pedagogical reality for the child with ASD and blindness, is generally left to the individual teacher to figure out.

While many aspects of teaching children with ASD may be problematic for children who are blind, the opposite also applies. There are approaches traditionally used for children with blindness that might not work well if the child also has ASD. For example, multisensory work can be difficult, since the child with ASD might have difficulty integrating information from more than one sense and experience sensory overload (Watanabe and Rees 2016). Also, hand-on-hand guidance (where the child’s hand rests on the adult’s, and follows its movements, making it possible to approach objects through tactile guidance) depends on the child’s appreciation of co-activity, which is not to be taken for granted when the child has ASD (Jordan 2005). Ideally, an effective educational program for a student with ASD and blindness should be based on the unique needs and abilities of the individual student and consider the core curriculum for both disabilities (Gense and Gense 2005).

In Sweden, most students with blindness or VI attend inclusive education, as do students with blindness and autism and/or other disabilities. A small number attend a national special school for students with multi-disability and visual impairment (MDVI). If a child has a diagnosed ID, they have the right to apply for a school or class especially for children with ID, or they can attend ordinary school with an adapted core curriculum. Consequently, students with autism but without ID, attend either regular classes in local schools, the national special school for students with MDVI or, sometimes, teaching groups for students with ASD. There is no expanded or adapted core curriculum for these students. Pedagogical support is provided to the schools by the Swedish National Agency for Special Needs Education (SNASNE or SPSM, Swedish abbreviation) and its two national resource center units. Additional support is provided by local low vision clinics and habilitation centers. However, the regular teachers together with paraeducators or teacher’s assistants, handle the everyday practice.

The aim of this study was to describe experiences of the school situation and the support for a sample of Swedish children with blindness and ASD. This paper focuses on the pedagogical situation and challenges as well as successful strategies in the schoolwork.

## Method

The study had a qualitative research design with an interview-based approach including students’, teachers’ and parents’ perspectives. In addition, pedagogical documentation was retrieved from the schools.

### Participants

The study included a strategically selected sample of children with congenital or early infancy blindness (defined as total blindness or light perception at the most) and ASD-diagnosis, as assessed and diagnosed according to DSM-IV (APA 1994). Based on medical records and psychological assessments, available from a previous population-based study of Swedish children with blindness (de Verdier et al. 2017), a total of 22 children between the ages of 7 and 16 (i.e. school age) were identified as matching the study’s inclusion criteria of blindness and ASD. Invitation letters were sent to eight of these 22 families. The selection was performed to include children of different ages, from primary to secondary school in addition to maintaining an even gender distribution and rural and urban representation. Moreover, the sample consisted of children both with and without concomitant ID and with school placement in inclusive as well as specialized settings. The purpose was to attempt to reflect the heterogeneity of the population, and capture experiences from children with blindness and ASD in different contexts. Of the eight invited families, one declined participation, and one did not respond. Thus, informed consent was received from six families. Invitation letters were then sent to the children’s schools, addressed to the teacher who knew the child best, according to the parents. The final participants were three girls and three boys between 9 and 15 years of age, from different parts of Sweden (Table 1).

**Table 1:** Demographic characteristics of the participating students

Student	1	2	3	4	5	6
Age	9	15	15	11	15	13
Etiololgy of VI	LCA	LCA	ROP	LCA	Anoph	Anoph
Type of ASD	Autism	Autism	Asperger syndrome	High functioning autism	Autism	High functioning autism
Cognitive level	Severe ID	AIF	AIF	AIF	Mild ID	BIF
Current school place-ment	Special school for students with ID and MD, small group	Special school for students with MDVI, small group	Small group for students with ASD/special needs located within regular school	Inclusive education, regular large class, support from para-educator	Special school for students with MDVI, small group	Inclusive education, regular large class, support from para-educator

*VI* Visual impairment, *LCA* lebers congenital amaurosis; *ROP* retinopathy of prematurity, *Anoph* anophthalmia, *ASD* autism spectrum disorder; *ID* intellectual disability, *AIF* average intellectual functioning, *BIF* borderline intellectual functioning, *MD* multi disability, *MDVI* multi disability and visual impairment

Only one child among the final participants had severe ID. Regarding cognitive level, the sample is thus not entirely representative for the total population of children with blindness and autism, where the proportion of children with moderate to severe ID is in fact larger. However, we prioritized inviting children with cognitive and linguistic abilities that would enable them to take part in an interview. In addition, the study included eight parents—one parent per child in four cases, and both parents in two cases, as well as seven teachers—one teacher per child in five cases, and two teachers in one case. Since the study group is small and might be easy to identify, considerations have been made to protect the identity of the participants. For this purpose, specific details about the students, families and schools have been omitted.

### Procedure

Qualitative, semi-structured interviews (Corbin and Strauss 2008) were performed with five out of six students (the student with severe ID could not participate in interview), the teachers and the parents. The interview guides were sent out in advance to all teachers and parents. The areas covered in the interviews concerned the organization of the student’s school situation, education and support to teachers, the student’s achievement in school, and challenges and successful factors in the schoolwork. The students’ interviews focused on their experiences of the school situation and the support they received. The five students received a letter in Braille prior to the researcher’s visit. The letter offered a simple description of what was going to happen during the interview as well as the researcher’s e-mail address and telephone number if the child had questions. The parents could choose whether one or both of them should participate in the interview, as well as the location for the interview (their child’s school, their home, the researcher’s office, and in one case over the telephone). Four out of five students were interviewed in their school, with the fifth being interviewed at home. All teachers were interviewed in the schools. The mean duration for the interviews with the students was 30 min, for teachers 69 min and for parents 76 min. All interviews were audio-recorded. The researcher also participated in the students’ classes prior to the interviews, in order to see the environment and allow the student to become acquainted with the researcher. During the visits to the schools, school grades and the teachers’ pedagogical documentation, such as individual evaluation plans (IEP:s), was collected for each student.

### Data Analyses

School grades, IEP-goals and other documentation of school achievement were compiled for each student, in order to acquire an overview of the students’ general achievement levels and progression in school. The audio-recorded interviews were transcribed, resulting in a total of 228 single spaced pages of written material. Interview data regarding facts about the students’ school history, academic achievements and teachers’ education, were summarized. The main parts of the interviews were then analyzed thoroughly through inductive thematic analysis (Braun and Clarke 2006). Thematic analysis is a qualitative descriptive approach, within the same tradition as descriptive phenomenology or content analysis (Vaismoradi et al. 2013). Methods following this tradition employs a lower level of interpretation than for example grounded theory or hermeneutic phenomenology. In descriptive methods such as thematic analysis, coding categories are derived directly from the text data, instead of taking its’ starting point in a specific theoretical assumption. Such methods are suitable when the aim is to describe a phenomenon and the existing research literature or theory is limited (Hsieh and Shannon 2005). The value of qualitative description lies, among other things, in the increased knowledge about experiences and phenomenon that can originate from the narratives of the respondents (Vaismoradi et al. 2013).

Thematic analysis has many similarities with conventional content analysis. However, while content analysis focuses more on the frequency of occurrence of categories and themes, by using a descriptive approach in both coding and its interpretation of quantitative counts of the codes, thematic analysis instead focuses on providing a more detailed and nuanced account of the data, involving the identification of central themes without quantification (Braun and Clarke 2006; Vaismoradi et al. 2013). In thematic analysis the relevance of a theme does not necessarily depend on quantifiable measures, but rather on whether the theme captures something that can be considered important in relation to the area of research (Braun and Clarke 2006). In the current study, this approach was chosen because we considered it to suit the purpose of the study best; regarding the aim to describe subjective experiences of a small and unusual study group.

The analyzing process included several steps (Braun and Clarke 2006). The first step involved the research team reading all the data (interviews with students, teachers and parents) several times, in order to become familiar with the contents. This was followed by the generating of initial coding of statements across the entire data set (all three informant-subgroups). In the next step the codes were combined into potential themes, which were visualized in a thematic map for each informant sub-group. The themes were then reviewed all over again, resulting in some of them being pooled into larger themes. In the last step the final themes were re-analyzed, defined and named. Finally, a thematic summary was developed, in which the themes were organized within two major, overarching areas; *Difficulties and challenges in the schoolwork* and *Successful strategies in the schoolwork*. The final analysis and summary was agreed by the members of the research team.

## Results

First we present a brief summary of the students’ school history, the educational levels of the teachers and the students’ general school achievement, based on information from the interviews and the documentation that was collected from the schools (teachers’ pedagogical documentation, IEP:s and school grades). Thereafter follows a description of the themes that were identified in the interviews, within the two areas of challenges and successful strategies in the schoolwork.

### An Overview of the Students’ School History

One student, with severe ID, started in a school for children with ID and multiple disabilities (MD). The other five students all started school included in regular classes, each with support from a paraeducator or teacher’s assistant. However, at the time of the study, only two out of five still remained in regular inclusive settings. The other three (one with mild ID, two with average intellectual functioning, AIF) had transferred to specialized settings at the secondary level. The reason for this was, in all three cases, that the regular schools had difficulty meeting the students’ needs, according to the parents. Two of these students (one with mild ID, the other one with AIF) now both attended the national special school for students with MDVI. The third student (with AIF) currently attended a group for students with ASD or other special needs, within a regular school. All the students in specialized settings, attended small classes with a high degree of staffing. The two students who were still in inclusive settings both attended large classes with full-time support from paraeducators (Table 1).

### Teachers’ Education

The basic educational level of the responsible teachers, paraeducators and other staff working with the students varied from special needs teacher to no pedagogical education at all. Most paraeducators and teachers had attended courses about teaching methods for students with blindness, and had received pedagogical support from SNASNE and support from low vision clinics. Some had also attended lectures or courses about autism. Most of them expressed that although the support they had received was helpful, it was insufficient, and was sometimes experienced as fragmented. None of them had received any formal education about the combination of blindness and autism. In the special school for students with MDVI, the teachers had considerable experience of students with blindness and autism or other additional disabilities. For the teachers in the other schools, this was a completely new experience.

### General School Achievement

Five out of six students were Braille readers; the student with severe ID had not developed reading skills. Two students had good decoding skills and reading speed, while three were considered slow or very slow readers, according to teachers’ descriptions and reading evaluations. All five students had problems with reading comprehension. Apparently, reading Braille on paper for some of them included many distractors—for example the spiral binding of the book, the feel of certain letters and the sound of turning the paper—which made it even more difficult to concentrate on understanding what they were reading. The students all expressed that they preferred audio books, because it was faster and easier to understand when you did not have to engage in tactile reading. Subsequently they all used a combination of audio and Braille in their computer.

The students mentioned some favorite school subjects, for example, language studies and crafts, but most of them liked almost all subjects. However, the outstanding favorite for all of them was music. Math was the subject least appreciated, because it was considered difficult and boring. According to the pedagogical documentation from the schools, at the time of the study all the students—with support—met the expected requirements in all subjects (with one occasional exception in math) according to the curriculum they studied (regular curriculum or curriculum for students with ID). The students at secondary level received grades on at least the lowest passing level.

### Themes Concerning Challenges and Successful Strategies

In each informant-group (students, teachers and parents) a number of themes were identified in relation to the two overarching areas *Difficulties and challenges in the schoolwork* and *Successful strategies in the schoolwork*.

### Difficulties and Challenges in the Schoolwork

The following themes were identified within the *Difficulties and challenges*-area (Table 2):

**Table 2:** Overview of the themes in each informant-group, related to difficulties and challenges in the schoolwork

Students	Teachers	Parents
Confusion	How to broaden the student’s horizons	Possible underestimation of the child’s capacity
Handling the surrounding stimuli	Balancing the need for individual support with group activities	The right choice?
	Motivation and study technique	
	Evaluation	

The themes in each informant-group will be presented and illustrated by quotes from the interviews. To protect the anonymity of the informants, quotes are not linked to specific individuals, but are instead labeled for example “student in regular class” or “teacher in special school”.

### Students’ Perspectives

The students’ experiences of main difficulties and challenges during the school day were expressed in two themes: *Confusion* and *Handling the surrounding stimuli*.

### Confusion

The students gave many examples, from both current and previous schools, of situations causing confusion. For instance, when teachers or classmates made incomprehensible statements: “They should think ahead and not say things that are wrong, or things they don’t really mean, otherwise it gets really confusing for me” (student in special school). Without the visual input to help clarify such situations, it was often difficult to understand. The visually based language used by some teachers led to additional confusion, and could cause feelings of exclusion: “They tell everyone to ‘look here’ and ‘look there’… but I can’t look so I don’t understand what they mean. It’s bad when they say that” (student in regular class). Furthermore, unstructured tasks or too much responsibility left to the student was difficult to handle: “The teacher needs to tell me exactly what to take out and what to do, otherwise I don’t know where to start” (student in regular class). It was also perceived as difficult and stressful when things were going too fast. One student expressed that: “it’s difficult when you have to think fast and answer fast and do things fast all the time” (student in regular class). Apparently, for the students who currently attended, or had previous experience of being part of a large class, this was a major problem. Things often happened too fast and the students missed the visual information, which led to confusion and insecurity.

### Handling the Surrounding Stimuli

The students stated that some of the most demanding aspects of the school environment were distracting noise, when many things were going on or when other students disturbed them when they were trying to focus on a task. All of them expressed that noise and bustle generally had a very negative effect on them and lead to difficulties in sorting out stimuli, making it hard to concentrate. One student described that “If it’s not calm, sensible and quiet, I just cannot think!” (student in special school). “One bad thing with my school is when the other children disturb me”, a student in a regular class expressed. For students who attended smaller groups this was not as big of a problem as it was for those in larger classes. One student, who had transferred from a large class to a small group, stated that: “Nobody should shout, but speak in a low tone of voice and be quiet if the teacher says so. I don’t understand why students don’t do as the teacher says—it’s so much easier if everyone just listens to the teacher. In my new class they do.” The general impression was that handling distracting surrounding stimuli took a great deal of energy during the school day, for all the students.

### Teachers’ Perspectives

Four themes were identified regarding challenges for the teachers: *How to broaden the student’s horizons, Balancing the need for individual support with group activities, Motivation and study technique* and *Evaluation*.

### How to Broaden the Student’s Horizons

Some of the students were very reluctant to try new things or do certain activities, according to the teachers, who struggled to encourage and motivate them. All teachers described these “thresholds” as preventing the students from using and developing their skills in different areas: “Well, [s/he’s] happy doing what [s/he] is used to do, but that doesn’t lead [her/him] forward…” (teacher in special school). The students displayed major difficulties with generalizing skills from one area to another, which contributed to their behavioral repertoire being limited. Much effort was put into trying to help the students to understand the use of skills in different situations in “real life”: “It is important that [s/he] understands how [s/he] can use skills [s/he] learns in school, outside school—in other contexts, in reality, so to speak. This is top priority!” (teacher in small group). Some of the students were also sensitive to changes and had difficulties handling when something did not turn out as expected. This could cause considerable distress and require a great deal of energy: “…[s/he] gets very worried and wants the mistake to be corrected immediately, otherwise [s/he] dwells on in for a long time, unable to think about anything else” (teacher in special school). The teachers expressed a wish to help the students develop their ability to make sense of the world and broaden their horizons. The students’ blindness contributed to their perception of the surrounding world as being restricted, and their autism caused additional problems with respect to understanding different contexts. One teacher in regular school concluded: “Well, [her/his] world is so small…[s/he] needs so many experiences, and help to understand how everything fits together. Only one disability would be challenging enough, in this respect…”

### Balancing the Need for Individual Support with Group Activities

Most of the students needed close support from an adult, and some of them clearly learned best when working alone with the teacher, free from other distractions. However, the teachers all had the ambition that the students should also be engaged in activities together with peers. One teacher in a special school described that: “[s/he] prefers working alone, but we try to arrange at least some activities together with classmates, with the aim to practice interaction… we think that’s important”. In a few cases, the teachers felt pressured by the parents to ensure that the student spent as much time as possible together with peers: “I know that [s/he] learns much more when I’m alone with [him/her], and [s/he] also prefers that. But still, we sometimes stay in the classroom, in the name of inclusion…” (teacher in regular class). This presented a dilemma, which was not always easy to handle in practice, especially regarding the students in larger classes. Some teachers hoped that group activities in the classroom would pave the way for interaction in other situations, but this was not to be taken for granted: “…group activities during lessons doesn’t really lead to any increased peer interaction in other contexts, such as break time” (teacher in special school). Instead, the students often ended up listening to music on their phone, or talking to an adult. It was often difficult to introduce activities that engaged both the student with blindness and ASD and the other students, because: “they have very different interests” (teacher in regular class). However, positive results also occurred: “Organized group activities has definitely increased [her/his] interest in other children” (teacher in special school).

### Motivation and Study Technique

All teachers stated that their students liked to learn and easily learned things that caught their interest. However, motivating and piloting the students forward was not always easy. Some of the students, who had transferred to new schools, had previous experiences that presumably affected their general motivation. A teacher in a special school described that: “Some of our students come from total failures in their local schools. Unfortunately, that’s often the main reason why they come to us. Therefore our job is to make school fun and increase the student’s motivation and self-esteem”. Furthermore, regardless of the different cognitive levels of the students, a common challenge was that they exhibited problems with executive functions, they had a slow tempo and were not very independent: “[S/he] has no inner drive. [S/he] neither knows how to begin, nor complete a task independently—you have to push the ‘start button’ all the time” (teacher in regular class). The teachers had many concerns about how to improve the students’ study technique and thereby increase their independence and ability to take initiatives and complete tasks: “We have to put a tremendous effort into trying to increase [her/his] ability to take initiatives” (teacher in small group). In this context, the teachers pointed to the need for more knowledge about how to adapt their teaching methods to suit the students’ needs from both the VI- and ASD-perspective.

### Evaluation

Some of the teachers expressed an insecurity concerning how to evaluate the students’ achievements properly: “Evaluation and grades… that’s challenging. I wish I had colleagues with more knowledge about these disabilities, to discuss this matter with” (teacher in regular class). For the teachers who had no prior experience of blindness and ASD, it was difficult to know what to expect from the student, and whether the student should be evaluated in the same way as the classmates. “It’s difficult to know whether you should compare this student to the other students at all, or only compare [her/him] with [her/himself]” (teacher in regular class). Sometimes they experienced that the challenges concerning how to handle the overall situation for a student with such complex disabilities were so overwhelming, that pushing the student beyond the “average-level” felt impossible. One teacher in regular class expressed that “I think [her/his] grades must be considered good enough, bearing in mind [her/his] difficulties”. A couple of teachers stated that they did see more capacity within their student, yet felt that they lacked the necessary competence to bring this capacity to the surface: “I think we could bring more out of [her/him], if we had better skills” (teacher in small group).

### Parents’ Perspectives

The parents did not have the same insight in specific challenges in the teaching situation, as the students and teachers. Their main worries instead concerned overall questions regarding their children’s possibilities to achieve at their best, and the difficulties with finding an optimal school placement. Two themes were identified in their interviews: *Possible underestimation of the child’s capacity* and *The right choice?*

### Possible Underestimation of the Child’s Capacity

All the parents had ambitions concerning their children’s possibilities to achieve and develop their skills as much as possible, yet they realized that there were many challenges in the work with their children, which really tested the teachers’ creativity. Some parents expressed concerns that these challenges lead to too low expectations regarding what the child could achieve. “It easily becomes a situation where they’re impressed that [s/he] does anything at all, sort of… which means that they don’t expect much…” (parent of child in special school, about the situation in former, regular setting). Some teachers’ lack of experience of children with similar disabilities, could involve a risk that the child did not move forward in their development, as much as they could: “I think the demands sometimes are set way too low because they don’t know what to expect of a child like this, and then nothing happens” (parent of child in regular class). Another parent of a child in regular class, were concerned about the grades not reflecting their child’s capacity: “[S/he] has many skills, but this doesn’t show in [her/his] grades. [S/he] is blind and has autism, and therefore needs help to perform in school. With help, the skills should be reflected in the grades”. The worry about possibly insufficient support and underestimation of the child’s capacity, for some parents lead to a constant feeling of stress: “I feel we have to be ‘on our toes’ all the time, we can never relax…” (parent of child in regular class).

### The Right Choice?

One theme that occupied the parents’ thoughts was the choice of school placement. Was there an optimal setting, and what did it look like? Their experiences and opinions of different school settings varied. Some parents argued in favor of special schools: “The special school definitely suits us best. I think [s/he] would get hurt in a regular class. Everyone would think that [s/he] is odd, when [s/he] is just being who [s/he] is…” (parent of child in special school). Others regarded inclusive education as the best option: “We definitely want our child to continue in inclusive education. We think that’s necessary, to prepare [her/him] for living a life as normal as possible” (parent of student in regular class). Some were more ambivalent, and had experience of different settings: “I must say that I’m really happy that [s/he] had those years in a regular class, even if it wasn’t perfect. Because that gave [him/her] many important experiences. And in the beginning I was convinced that inclusive education was ‘the thing’. But right now I’m incredibly happy that we moved [her/him] to a small group where everyone has… needs. I won’t say that one option is better than the other, but for our child, senior level in a regular class would have been complete chaos, and I think that [s/he] would have been very lonely” (parent of child in small group). General advantages and disadvantages with inclusive education and specialized settings was a topic that engaged all the parents: “One advantage with a smaller, specialized setting is that it’s less chaos and more adapted teaching…. Easier to learn stuff” (parent of child in special school, who previously attended a regular class). A parent of a child in regular class, who was hesitant regarding whether this was the optimal choice, said that: “I’m really for smaller contexts, definitely. Where the kids are more alike, and they can find friends with whom they can identify, instead of always feeling different”. But the risk of special schools leading to exclusion from the regular society was also pointed out: “…the advantages with a regular class is that you’ll be more prepared for taking part in ‘normal’ contexts, which you will inevitably encounter later… (…) A specialized group or school becomes more… closed, in a way… excluding. Limited topics of conversation, a limited view of the surrounding world…” (parent of child in special school). The parent of a child who had gone through several school transitions and currently attended a special school, made the following conclusive remark: “I don’t think there’s any right or wrong… I think It’s really important that inclusive education exists, for those children who are able and comfortable with that. But I don’t think you should be afraid of specialized solutions either. I’ve certainly seen that my child needs that. [S/he] can’t handle being in a regular class, that’s much too chaotic for [her/him]. And all teachers can’t have the same competence. So I think there must be different choices”. In summary, the parents’ search for an optimal school placement involved many questions, to which there seemed to be no simple answers: “We’re constantly looking for answers… what’s the best option? But we don’t get any answers.” (parent of student in regular class).

### Successful Strategies in the Schoolwork

The following themes were identified as related to the successful strategies-area (Table 3):

**Table 3:** Overview of the themes in each informant-group, related to successful strategies in the schoolwork

Students	Teachers	Parents
Peace and quiet	Small context and flexible working forms	The attitude from teachers
To understand and to be understood!	Planning, structure and control	Address individual needs and differences
	Building on strengths and interests	

The themes in each informant-group will be presented and illustrated by quotes from the interviews.

### Students’ Perspectives

The students’ opinions about what was important and helpful in their schoolwork were summarized in two themes: *Peace and quiet* and *To understand and to be understood*.

### Peace and Quiet

A calm working space with a moderate sound volume, was pointed out by the students as immensely important. The opportunity to withdraw from the bustle in the classroom was sometimes necessary, especially for the students who attended larger classes: *“*When I want to work on my own in a separate room I should be allowed to do so, because it means that right now I really need to be on my own and focus on what I’m doing” (student in regular class). The students who had changed schools, could compare the different learning environments and point to improvements related to the size and atmosphere of the class: “It’s easier when there are not so many students in the class, I don’t become so stressed anymore”, one student who had transferred from a large class to a smaller group described. Another student, who had transferred from a large class to a special school, said that one of the greatest improvements was that: “… it’s calm, finally. (…) The sound volume is much lower; you get the chance to concentrate much better. (…) In my old school it was much noisier; the children shouted and then the teachers shouted at them to be quiet…” In summary, all the students talked about the importance of reducing the sound level, and stated that peace and quiet was their number one characteristic of an optimal learning environment.

### To Understand and to be Understood!

“Well, simply, the teacher must learn how to teach students just like me. It’s as easy as that!” one student in regular class said, when asked to describe good teaching. Activities and tasks should be arranged in a manner that made it possible for the student to understand what was happening and what they were supposed to do. One student (in special school) stated that: “Teachers must be structured and clear when they talk and teach. Otherwise I don’t understand what to do”. To be understood by the teachers included being treated in the same way as the other students in the class. The students in regular classes resisted when teachers pointed them out, for example by making a big deal out of giving them separate materials or special treatment in front of the peers. One student, who had moved from a regular class to a small group for students with special needs, felt a relief to be with others who also needed extra support: “I like to be with other students who also need extra help, we understand each-other better. It feels good that I am not the only one.” Regarding working forms, even though some of the students preferred working independently with tasks, “I mostly enjoy working on my own, I get more done” (student in regular class), it seemed important not to assume that this was always the preferred way of working. In fact, some of the students expressed that they gladly took part in group activities, as this made them feel like part of the class: “I enjoy working in groups, because that makes me feel participatory” (student in regular class). However, to make these activities into positive experiences, it was crucial that they were arranged so that all students in the group could understand, participate and contribute. Otherwise too much energy had to be put in to trying to understand the often fast and visually based communication between the students, grasp what was going on and what was supposed to be done: “It has to be clear what I am supposed to do, and what they are supposed to do” (student in a small group). Also, it was helpful if the student could perform a task they mastered well. In this way, they could more easily understand, make themselves understood and contribute in a positive way.

### Teachers’ Perspectives

Despite the fact that many of the teachers stated that they lacked enough competence about blindness and ASD, they had many ideas about contextual factors and practices that benefited the students’ learning. Three themes were identified in this area: *Small context and flexible working forms, Planning, structure and control* and *Building on strengths and interests*.

### Small Context and Flexible Working Forms

When asked to describe an optimal learning environment for their student, all teachers described that a small, calm group with a quiet, friendly atmosphere, was the ideal starting point. One teacher in a regular class made the reflection: “This class is far too large for [her/him]. It is absolutely not optimal. (…) You can tell [s/he] doesn’t stand a chance to get an overview of what’s happening”. Depending on the class size, the atmosphere, and the task, the teachers described that different working forms needed to be applied in a flexible manner. Also, some of the teachers stated that their students’ mood very much affected their functioning, which contributed to the need for flexibility: “We must have the opportunity to arrange activities in different ways depending on both the subject and the student’s mood of the day. If it’s a bad day nothing works, if it’s a good day almost everything works. Therefore, we need to be flexible” (teacher in special school). It was considered necessary to have access to a smaller room near the classroom in order to arrange possibilities to work in different settings; individually or in small constellations. For some students pair-activity was the best option, since “group activities with 3–4 children are too difficult. [S/he] totally disappears” (teacher in regular class). A common arrangement was that the student initially took part in the introduction of a task together with the classmates in the classroom, and then moved into a neighboring room to complete the task in peace and quiet.

### Planning, Structure and Control

Generally, activities that worked well for the students were characterized by a high degree of planning and structure, and a low degree of improvisation, according to the teachers. Planning included both overall planning of the day and the detailed planning and adaptation of specific tasks and tactile materials. The students needed to be prepared in advance, before introducing a new task or activity: “My student has a tremendous need to have a sense of control and to know what is going to happen” (teacher in special school). All the teachers gave examples of successful strategies and methods applied, when preparing and introducing tasks. These are presented below as a “think tank” of pedagogical ideas, building on preparation and structure:

- Find out which sensory channel works best for the student, so that information can be provided in an optimal way
- Avoid sensory overload by not providing too much information through multiple senses
- Provide pre-understanding of the area that is subject of the task, and let the student explore new material thoroughly through their preferred sensory channel
- Divide the lesson into parts and define exactly what will happen in each part
- Provide a list of all the elements the student is supposed to perform during the lesson, including what material to take out and what to put back or clean up
- Make sure material is placed in a location where the student can easily find it
- Provide a clearly defined frame and structure for how to perform the task, and re-use this frame in other tasks so the student becomes familiar with it
- Provide a clear beginning and end of each task
- Provide a detailed instruction of each specific element in every task
- Limit the amount of items within the task
- Limit the amount of information; “stick to the important stuff—remove all the ‘fluff’”
- Provide a clear definition of how long the student shall work with the task
- Provide the possibility to take micro-breaks if needed
- Provide multiple choice-questions when discussing the contents in texts
- Continuously go back and check that the student has understood, repeat steps if necessary
- Use the same design and instruction for homework as in school, and teach parents how to support their child

How to present instructions, lists and working schedules in the best way varied between the students. Some had tried tactile schedules with objects or tactile graphics. However, if the student had grasped the verbal concepts, they generally preferred other means, for example Braille and/or audio recording.

### Building on Strengths and Interests

One way of motivating the students was using their strengths and interests in tasks and activities. If used creatively, the students’ special interests could be the starting point in many school subjects: “[S/he] is very technically interested, and this opens up many opportunities to motivate [her/him] in the schoolwork” (teacher in special school). One area that all the students had in common was their interest in music. All the students were described as talented in music; some of them even exceptionally gifted, with absolute pitch. Music could be used for many purposes: enjoyment, interaction with peers, relaxation, as a reward, as a means for theoretical learning and as a way to create meaningful break activities. Through their musical skills the students were also given the opportunity to shine among the peers: “We try to use music as much as possible. Music really brings out the best in [her/him]!” (teacher in regular class). Also, the teachers hoped that the students could make use of their strengths and interests in the future, if given the chance to develop them as much as possible during school: “If [s/he] can continue developing [her/his] talents in music, that might help in achieving a meaningful occupation later, after school. It is not easy to know what is possible for [her/him] to do in the future, but I really hope that [s/he] could do something that involves music, so [her/his] talent is cherished” (teacher in special school).

### Parents’ Perspectives

In the parents’ interviews, two themes were identified when discussing successful strategies: *The attitude from teachers* and *Addressing individual needs and differences*.

### The Attitude from Teachers

All parents agreed that their children were dependent on structure, control and familiarity to function optimally. They stressed the importance of making tasks fun and meaningful in order to increase motivation and independence, and promote development. To achieve this, the teachers’ attitude was regarded as a key factor. Both positive and negative experiences of teachers’ attitudes were described. Some had bad experiences from previous schools: “In the old school [s/he] was discriminated. They had a really bad attitude; we don’t think they wanted [her/him] in their school” (parent of child in regular class, about the previous school). Another parent described that: “As a parent, to feel like no-one wants my child… that’s about the worst feeling you can experience” (parent of child in special school, about experience from regular setting). This parent experienced great relief when meeting engaged teachers in the current school: “They are passionate about working with these children. They have actively chosen to work with children like mine, and that feeling was a completely new experience for me” (parent about the current special school). For the students in regular classes, who encountered many challenges as being the only student with disability in the class, the teachers’ attitude meant everything for a positive outcome: “So far [s/he] has tagged along with the others, all thanks to a very engaged teacher… (…)[S/he] is gold. Since we have [her/him], we can feel a little more relaxed” (parent of child in regular class). Another parent expressed: “If there’s a will, the right help is created.” (parent of child in regular class). In summary, one parent concluded: “The attitude from the school management and the teachers spread to the children, so it means everything” (parent of child in regular class).

### Addressing Individual Needs and Differences

All the parents pointed out the importance of addressing each child’s individual needs, and strive to bring forward their unique strengths and talents. They were aware that this put great demands on the teachers, and some parents reflected on the fact that teachers in regular schools had, in many ways, an impossible assignment: “In a regular class the teacher is expected to be able to work with all types of children, whether they like it or not. But it’s impossible to have sufficient knowledge about all types of learning disabilities, isn’t it? So the concept of ‘one school fits all’… that’s totally unrealistic” (parent of student in regular class). The parents regarded it as very important that their children were allowed to “be themselves” and that their differences were embraced, regardless of type of school placement. Their children should have the same rights as everyone else, and this required competence. “Well this is my child—take it or leave it. [S/he] is the way [s/he] is, and [s/he] must have the same opportunities to develop as any other child” (parent of child in special school). One parent concluded that: “Our children are different, and with different needs. You have to see to each individual, and continuously evaluate the school setting and the teaching methods. (…) Sometimes [our child] amazes us with what [s/he] can do—if [s/he] is just given the chance” (parent of student in special school).

## Discussion

This study elucidates pedagogical challenges and dilemmas concerning a small and complex group of students. We considered it valuable that the students’ themselves were given the opportunity to be heard through the qualitative method that was adopted in the study. The students’ voices, in addition to teachers and parents, provide important insight into the complexity of these students’ school situation. Our study group is not entirely representative for the population of children with blindness and ASD, but was selected to represent different severities of ASD, with or without concomitant ID, and different types of school placement.

### Pedagogical Understanding of the Executive Functioning Deficits

Both teachers and students clearly described specific problems related to the students’ apparent deficits in executive functioning, regardless of their general cognitive level. Specifically, the need for help with the structure and planning of school assignments, motivation, starting and completing schoolwork along with difficulties related to situations requiring flexibility and sufficient processing speed, emerged in the interviews. The students’ also pointed specifically to their great need of peace and quiet in the learning situation, to be able to focus and concentrate. One might speculate that some of the students would also meet criteria for attention-deficit/hyperactivity disorder (AD/HD), predominantly of the inattentive subtype, in addition to ASD. However, this had not been especially assessed. At school and also in other situations it is necessary to consider these cognitive deficits, and specific educational help has to be provided.

Many of the successful pedagogical strategies that were described in the interviews applied the principles of common practices for ASD and other neuropsychiatric conditions. The foundation in the approaches that were used seemed to stem from challenges related to ASD or other neuropsychiatric difficulties, rather than blindness. The interviews confirmed that the autism was considered a much greater challenge than the VI. However, in order to make use of the ASD-related principles, knowledge about the restrictions related to the lack of vision is also necessary. Thus, an assessment of the student’s primary sensory channel/s for learning and communication should always be performed, so that information and instructions can be presented in a manner that suits the student and enables her/him to understand and respond (Gense and Gense 2005).

Altogether, the pedagogical strategies described in this study are in accordance with the literature about ASD and VI concerning the need for structure, schedules, work systems and routines (Howley and Preece 2003; Gense and Gense 2005; Jordan 2005; Odom et al. 2010). In addition, Jordan (2005) emphasized the importance of creating a structure in the physical environment, in order to facilitate the students’ understanding of the functions of different areas and enable the student to navigate more independently. For example, tactile symbols can be used to mark different areas. If the student is given the symbol of a specific area and then is taught to navigate to find a matching symbol by the entrance to this area, this also contributes to priming the student for the type of activity that is to occur there. Gense and Gense (2005, 2011) pointed to the specific problems with generalizing which are accentuated through the combination of VI and ASD, and stressed the importance of developing a plan for providing opportunities to develop the student’s generalization skills. In summary, according to them, it is necessary that the evidence-based practices in the field of ASD are also applied for blind students, of course with adequate adaptations (Gense and Gense 2011).

### Improving the Support to Teachers

A general opinion among the teachers in this study was that they had received more education regarding blindness than regarding autism, despite the fact that the ASD-related difficulties were far more challenging. Also, formal education and support regarding the combination of blindness and ASD was practically non-existent. Generally, the teachers had to use their own creativity to combine the two perspectives in their teaching. Even though many of the teachers had found ways to apply strategies in their teaching that apparently were successful, the lack of proper education was considered by most of them a major problem, since it took a great amount of time and effort to plan and adapt methods and materials. Many of the teachers expressed a need for more hands-on support from someone with competence about the dual disability and concrete examples of how to design and adapt tasks. Furthermore, it was clearly an advantage when more than just one or two teachers in the school received education, since competence in the whole team increased the opportunities to work more flexibly and support each other in handling challenges. Thus, a more coordinated, continuous and concrete support to the entire team would be desirable. Also, the school management’s understanding of the students’ complex needs is necessary to create the time and conditions required in order to transform the support received, into pedagogical reality.

### The Optimal School Placement?

Five out of six students in our study started school in regular classes, but only two remained in inclusive education at the time of the study. The families’ experiences of their children’s school situation varied greatly, and finding an optimal school setting was not easy. Subsequently, the parents were occupied with the question of school placement, and whether they had made the right choice for their child. In one case, the search for an optimal placement had resulted in several school changes, each time leading to distress and backlash before the student could settle in their new environment. Since students with ASD are more sensitive to changes and in need of more continuity than most other students, situations like this are highly problematic. The parent of the student with severe ID was actually the one most satisfied with the school placement from the beginning and onwards. For this family it was obvious that a special school for students with ID and MD was the only option; the parent stated that the fact that their child had such extensive disabilities, actually made the choice of school easier. Had the child been on a higher cognitive level, the school options would have been more limited, since students with ASD and blindness, but not ID, are primarily referred to regular schools. In the special school, this student received an individually adapted support that suited the family well.

Previously, all Swedish students with blindness or VI, both those with and without additional disabilities, attended the state special school where the staff had specific competence about these students’ learning needs. Despite 30 years’ experience of inclusive education, the school system’s ability to seize and cater to the needs of students with blindness and complex additional disabilities such as autism, is still insufficient. Hence, the risk of underestimation of the children’s capacity and underachievement due to a lack of proper support is obvious—as both parents and teachers in this study pointed out.

There is probably no single “best” school placement for a student with blindness and ASD. However, some points are worth bringing forward. Jordan (2005) discussed the advantages of a learning environment with staff that have an expertise in VI, and where additional support in ASD is added. According to Jordan, this is more important when the student has blindness or severe VI, since in the reversed position, such as an ASD-environment where knowledge about VI is added, the teaching is likely to be very much based on the visual structuring of environment and activities. However, Howley and Preece (2003) pointed out that with knowledge, it should be possible to adapt visually based methods for other senses, and in reality this may be the most practical option. But what is said about inclusive education in regular classes for these students? Jordan argues that there should always be an aim for inclusion, but the basic learning should take place in a setting where there is maximal support from people who really understand the complexity of the student’s dual disability (2005). If the student attends a regular, inclusive setting, a transdisciplinary model of support with collaboration between the teachers, parents and specialists in VI and ASD is necessary (Jordan 2005). Also, regardless of the type of school placement, the dilemma concerning how to balance the students’ need for individual support with taking part in group activities, must be addressed. Gense and Gense (2011) suggests that students with VI and ASD should have an expanded core curriculum, that addresses their specific learning characteristics and needs, and puts the learning into a life-long perspective. It is a great challenge for educational research to find and evaluate methods that provide the most optimal learning environment for these students.

### Strengths and Limitations

The research about pedagogical solutions for students with blindness and ASD is very limited. Furthermore, studies that allow students’ voices to be heard are unusual. In this sense, this study may provide a valuable contribution to the field. However, the small scale and exploratory, qualitative approach of the study, also brings certain limitations. The results reflect subjective experiences of different contexts, and the sample was not randomized, but strategically selected. The reason behind this choice of sampling was that since the total population of children with blindness and ASD is very small, the number of children who could be invited, was limited from the start. However, including all of them in the study was not possible, due to practical considerations. Therefore, the participants were selected with the aim to reflect the heterogeneity of the total population rather than use randomization, to eliminate the risk of having an over-representation regarding some of the variables. By doing this, the participants’ experiences, even though subjective, hopefully elucidate areas that are relevant in the discussion about the pedagogical situation for this complex group of students.

## Conclusion

The results of this study imply that for children with the complex combination of blindness and ASD, with and without ID, there is a strong need for pedagogical solutions that take the students’ individual needs and abilities into consideration. To pinpoint what characterized successful teaching, one student humorously concluded: “Well, simply, the teacher must learn how to teach students who are just like me. It’s as easy as that”. However, in reality it is not that easy. The goal must be that each student is given the opportunity to develop their full potential, and use their skills and talents. To reach this goal, there is a need for optional school placements, where the students’ need for individual support can be combined with the possibility to be part of a social context with other students, according to their own wishes and abilities. Also, the students’ problems with sensory processing, possible cognitive overload and executive functioning, must be considered when shaping the school environment, and adapted, evidence-based ASD-practices should be applied. For this to be possible, more education for teachers concerning the combination of blindness and ASD, and the consequences for teaching and learning, is necessary. Increased collaboration between different actors within the support system, together with parents, is necessary in this process, as is the promotion of networks for knowledge exchange between teachers working with these students around the country.
